# Bearing Fault Diagnosis Method Based on Improved Singular Value Decomposition Package

**DOI:** 10.3390/s23073759

**Published:** 2023-04-05

**Authors:** Huibin Zhu, Zhangming He, Yaqi Xiao, Jiongqi Wang, Haiyin Zhou

**Affiliations:** 1 College of Sciences, National University of Defense Technology, Changsha 410073, China; 2 Beijing Institute of Spacecraft System Engineering, China Academy of Space Technology, Beijing 100094, China

**Keywords:** mode mixing, feature extraction, singular value decomposition package, signal decomposition, bearing diagnosis

## Abstract

The singular value decomposition package (SVDP) is often used for signal decomposition and feature extraction. At present, the general SVDP has insufficient feature extraction ability due to the two-row structure of the Hankel matrix, which leads to mode mixing. In this paper, an improved singular value decomposition packet (ISVDP) algorithm is proposed: the feature extraction ability is improved by changing the structure of the Hankel matrix, and similar signal sub-components are selected by similarity to avoid having the same frequency component signals being decomposed into different sub-signals. In this paper, the effectiveness of ISVDP is illustrated by a set of simulation signals, and it is utilized in fault diagnosis of bearing data. The results show that ISVDP can effectively suppress the model-mixing phenomenon and can extract the fault features in bearing vibration signals more accurately.

## 1. Introduction

As one of the key components of mechanical systems, the health of bearings is an important guarantee for their safe operation [1,2,3]. When a bearing fails, the bearing components form a sudden shock pulse through the fault site and cause the bearing and adjacent components to vibrate, so that the collected vibration signal contains the fault-specific periodic shock pulse signal [4,5]. Therefore, the bearing vibration signal contains important information about the health status of the bearing [6,7,8]. Therefore, in bearing fault diagnosis based on vibration signals, the core is to extract fault-specific signal features from vibration signals.

In the field of bearing fault diagnosis, in order to effectively extract the fault features in the vibration signal, existing research has proposed a variety of signal processing methods to be adopted to extract the fault signal features from the perspectives of the time domain [9,10], frequency domain and time–frequency domain, such as correlation analysis based on the time domain [11], Fourier transform based on the frequency domain [12], wavelet transform based on the time–frequency domain [13], empirical mode decomposition [14] and their derivative algorithms [15,16,17,18]. Although the above methods can effectively extract bearing fault features, correlation analysis and Fourier transform have difficulty effectively dealing with non-stationary and other complex signals. Wavelet transform needs understanding of the signal characteristics in advance to select the appropriate wavelet basis. Empirical mode decomposition has endpoint effects and model-mixing phenomena, which limit the use of the above methods to a certain extent.

Singular value decomposition (SVD) is also applied in the field of signal processing [19,20]. Ying et al. [21] realized the feature-space change of unknown source signals by SVD and then combined it with joint diagonalization to realize the separation of unknown source signals dominated by random noise. Golafshan et al. [22] applied SVD and the denoting process based on the Hankel matrix to the time-domain vibration signal of the bearing to eliminate the background noise and improve the reliability of the fault-detection process. Zhang et al. [23] applied SVD to signals under a sliding window and obtained a time-varying singular value matrix, which was used to enhance the identification of periodic faults. However, the traditional SVD algorithm is essentially carried out in the same resolution and hierarchical space when processing signal data [24], and it is difficult to effectively extract signal features. To solve this problem, Zhao et al. [25] proposed a multi-resolution singular value decomposition (MSVD) algorithm based on the idea of wavelet transform. The decomposition results of SVD are divided into different hierarchical spaces, and the signal of the bearing is decomposed into several approximate signals and detail signals with different frequencies by the SVD method. Then, in order to further improve the feature-extraction ability of MSVD, Zhao et al. [26] improved the MSVD and proposed a singular value decomposition package (SVDP), which shows that the method has the same decomposition structure as the wavelet packet and is suitable for extracting weak bearing fault features. Although MSVD and SVDP have been well-applied in bearing signal processing, Huang et al. pointed out that SVDP has mode mixing after multiple iterations; that is, a single sub-component signal contains completely different frequency components, or the same frequency component is decomposed into different sub-component signals. To solve this problem, Huang et al. proposed the extended singular value decomposition package (ESVDP) and the fast extended singular value decomposition package (FESVDP) to solve the model-mixing phenomenon and applied it to railway vehicle bearings [27]. However, the research in this paper found that Huang et al. did not completely solve the model-mixing phenomenon of SVDP.

To solve the above problems, this paper proposes an improved singular value decomposition package (ISVDP) algorithm to solve the model-mixing phenomenon in SVDP. On the one hand, ISVDP improves the feature-extraction ability of the algorithm by changing the structure of the Hankel matrix in SVDP. On the other hand, the similarity is used to select similar sub-signals, which further suppresses the model-mixing phenomenon. The content of this paper is as follows: In Section 2, SVDP and its improved algorithm ESVDP are introduced. In Section 3, an improved singular value decomposition package algorithm is proposed to overcome the shortcomings of SVDP and ESVDP algorithms in bearing feature extraction. Section 4 illustrates the effectiveness of the proposed method through simulation. Section 5 shows the comparison results of ISVDP and other methods on the bearing dataset, which verifies the effectiveness of the proposed method. Section 6 is a summary.

## 2. Singular Value Decomposition Package and Other Improved Algorithms

### 2.1. Singular Value Decomposition Package and Other Improved Algorithms

The purpose of a singular value decomposition packet (SVDP) is to use singular value decomposition to realize signal decomposition similar to that of a wavelet packet. SVDP can decompose the original one-dimensional bearing signal into a series of component signals of different layers by performing iterative singular value decomposition on the constructed two-row Hankel matrix, and feature extraction of the bearing fault signal can be realized [26]. The method of SVDP is as follows:

Let x(n) be a one-dimensional bearing signal, where n=1,2,⋯,N, *N* is the length of the signal x(n). The Hankel matrix of signal x(n) is constructed as

(1) $$H=\left(\begin{array}{cccc} x(1) & x(2) & \cdots & x(N-1)\\ x(2) & x(3) & \cdots & x(N) \end{array}\right)$$

Assuming that the J−1 layer SVDP decomposition has been performed, the $2^{J-1}$ signal $S_{J-1}^{i}=\left(x_{J-1,1}^{i},\cdots ,x_{J-1,N}^{i}\right)$ is obtained, where $i=0,1,\cdots ,2^{J-1}-1$ is the serial number of the sub-signal, and the Hankel matrix is constructed as

(2) $$H_{J}^{i}=\left(\begin{array}{cccc} x_{J-1,1}^{i} & x_{J-1,2}^{i} & \cdots & x_{J-1,N-1}^{i}\\ x_{J-1,2}^{i} & x_{J-1,3}^{i} & \cdots & x_{J-1,N}^{i} \end{array}\right)$$

**Figure 1:**
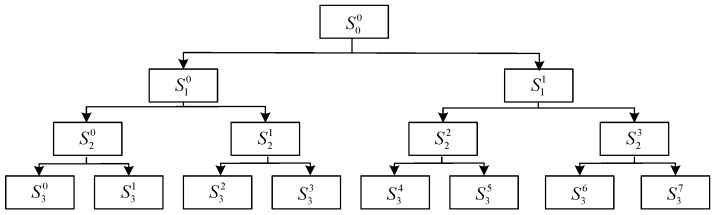
Schematic diagram of SVDP.

SVD is decomposed into

(3) $$H_{J}^{i}=U_{J}^{i}\Sigma_{J}^{i}{\left(V_{J}^{i}\right)}^{T}$$

where $U_{J}^{i}=\left(u_{J1}^{i},u_{J2}^{i}\right),U_{J}^{i}\in R^{2\times 2}$ and $V_{J}^{i}=\left(v_{J1}^{i},\cdots ,v_{J(N-1)}^{i}\right),V_{J}^{i}\in R^{\left(N-1)\times (N-1\right)}$ are left and right singular matrices, $\Sigma_{J}^{i}=\left[diag\left(\sigma_{j1}^{i},\sigma_{j2}^{i}\right),O\right]$ is a diagonal matrix and $\sigma_{J1}^{i},\sigma_{J2}^{i}$ are the approximate singular values and the detail singular values obtained by the $S_{J-1}^{i}$ decomposition of the *J* layer signal, respectively. The approximate matrix $H_{J1}^{i}$ and the detail matrix $H_{J2}^{i}$ corresponding to the singular values are expressed as

(4) $$\left\{\begin{array}{c} H_{J1}^{i}=\sigma_{J1}^{i}u_{J1}^{i}{\left(v_{J1}^{i}\right)}^{T}\\ H_{J2}^{i}=\sigma_{J2}^{i}u_{J2}^{i}{\left(v_{J2}^{i}\right)}^{T} \end{array}\right.$$

where $u_{J1}^{i},u_{J2}^{i}\in R^{2\times 1},v_{J1}^{i},v_{J2}^{i}\in R^{(N-1)\times 1}$. The diagonal averaging method in the singular spectrum analysis is used for the approximate matrix $H_{J1}^{i}$ and the detail matrix $H_{J2}^{i}$, respectively, to obtain the two component signals $S_{J}^{2i}$ and $S_{J}^{2i+1}$ of the signal $S_{J-1}^{i}$ at the *J* layer [28]. Using the above process, specifying the decomposition level *J*, SVDP can decompose the signal into $2^{J}$ sub-signals. When the number of decomposition layers J=3, the decomposition structure of SVDP is shown in Figure 1.

### 2.2. Extended Singular Value Decomposition Package

**Figure 2:**
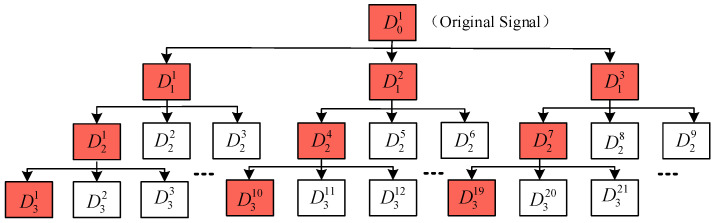
Schematic diagram of ESVDP and FESVDP (the red subcomponents).

Huang et al. pointed out that when performing SVDP decomposition on bearing fault signals with multiple resonance bands, there is obvious mode mixing between component signals, which reduces the extraction effect of bearing fault signal features. In addition, Huang et al. showed that as the number of rows of the Hankel matrix increases, the aggregation of singular values on the resonance band becomes worse, and for the singular values after the decomposition of the Hankel matrix, the sub-signal components corresponding to the adjacent singular values have the same frequency. Accordingly, Huang et al. improved SVDP by constructing an even-numbered Hankel matrix larger than two rows and merging the sub-signal components corresponding to adjacent singular values, and they proposed the Extended Singular Value Decomposition Package (ESVDP) and the Fast Extended Singular Value Decomposition Package (FESVDP) to improve the feature extraction ability of bearing fault signals; the methods of ESVDP and FESVDP are as follows:

Let x(n) be a one-dimensional bearing signal, where n=1,2,⋯,N, and *N* is the length of the signal x(n). The 2k-row Hankel matrix is constructed as

(5) $$A=\left(\begin{array}{cccc} x(1) & x(2) & \cdots & x(N-2k+1)\\ x(2) & x(3) & \cdots & x(N-2k+2)\\ \vdots & \vdots & \ddots & \vdots \\ x(2k) & x(2k+1) & \cdots & x(N) \end{array}\right)$$

where $k\in N^{*},k\geq 2$ is the number of sub-signals. Using the same method as in Section 2.1, 2k signal sub-components are obtained, and the 2k−1-th and 2k-th signal sub-components are combined to obtain the *k* sub-signal $D_{J}^{k}$, and then the above steps are repeated until the specified number of decomposition layers *J* is reached. The signal in the extended singular value decomposition package is decomposed into *k* sub-signals each time, and a total of $\left(k^{J}-1\right)/\left(k-1\right)\;$ sub-signals are obtained. Therefore, in order to reduce the calculation cost, FESVDP is proposed on the basis of ESVDP. FESVDP only retains the first sub-signal in each decomposition at the J=2 layer and later. When K=3,J=3, the decomposition structure of ESVDP and FESVDP is shown in Figure 2. The subcomponents marked red constitute FESVDP.

## 3. Improved Singular Value Decomposition Package

### 3.1. The Deficiency of SVDP and ESVDP in Bearing Feature Extraction

The core idea of SVDP is to construct a two-row Hankel matrix and perform singular value iterative decomposition on it. Therefore, SVDP can only get two singular values and the corresponding sub-signals at a time. Further, since the second-row vector of the Hankel matrix is only one datum later than the first-row vector, the two row vectors are highly correlated. At this time, the signals corresponding to multiple resonance bands are mainly concentrated in the approximate signals corresponding to larger singular values. Therefore, mode mixing occurs after SVDP decomposition [27].

The core idea of ESVDP is that the increase of the rank of the Hankel matrix makes the aggregation of singular values in the resonance band worse, and the purpose of separating multiple resonance frequency bands can be achieved by increasing the number of rows of the Hankel matrix. Further, another core idea of ESVDP is that the sub-signals corresponding to the two adjacent singular values have the same frequency, and the purpose of reducing mode mixing is achieved by superimposing the sub-signals corresponding to the two adjacent singular values. However, Treichler [29] pointed out that the Hankel matrix is composed of sine or cosine signals, and the singular value of the matrix is

(6) $$\sigma_{1},\sigma_{2}=\frac{a}{2}\sqrt{\left[L\pm \frac{\sin(L\omega T)}{\sin(\omega T)}\right]}$$

where $\sigma_{1},\sigma_{2}$ are the singular values of the matrix, *a* is the signal amplitude, ω is the signal frequency, *T* is the sampling interval and *L* is the number of columns of the Hankel matrix. According to Equation (6), one frequency corresponds to two singular values, but the two adjacent singular values do not always correspond to one frequency. Therefore, ESVDP does not completely solve the mode-mixing phenomenon existing in SVDP.

### 3.2. Improved Singular Value Decomposition Package

It can be seen from Section 3.1 that ESVDP does not completely solve the modal-mixing phenomenon existing in SVDP. Therefore, in order to solve the modal-mixing phenomenon existing in VDP, this paper proposes an improved singular value decomposition package (ISVDP). The algorithm is as follows:

Let x(n) be a one-dimensional bearing signal, where n=1,2,⋯,N; *N* is the length of the signal x(n). Construct the Hankel matrix as

(7) $$X=\left(\begin{array}{cccc} x(1) & x(2) & \cdots & x(L)\\ x(2) & x(3) & \cdots & x(L+1)\\ \vdots & \vdots & \ddots & \vdots \\ x(m) & x(m+1) & \cdots & x(N) \end{array}\right)$$

where N=m+L−1. Let L>m; through the SVD decomposition of Equation (7), the Hankel matrix *X* can be expressed as

(8) $$X=U\Sigma V=\sum_{i=1}^{m}\sigma_{i}u_{i}v_{i}^{T}=\sum_{i=1}^{m}X_{i}$$

where $U=\left[u_{1},u_{2},\cdots u_{m}\right]\in R^{m\times m}$ and $V=\left[v_{1},v_{2},\cdots ,v_{L}\right]\in R^{L\times L}$ are left and right singular matrices, and $u_{i},i=1,\cdots ,m$ and $v_{j},j=1,\cdots ,L$ are left and right singular vectors corresponding to singular values, respectively. $\Sigma =\left[\Lambda \;\;O\right],\Lambda =diag\left(\sigma_{1},\sigma_{2},\cdots ,\sigma_{m}\right)$ is a diagonal matrix, $\sigma_{1}\cdots ,\sigma_{m}$ is a singular value, *O* is a zero matrix, $X_{i}=\sigma_{i}u_{i}v_{i}^{T},i=1,\cdots ,m$ is a submatrix. Reconstruct the submatrix $X_{i}$ as

(9) $${\hat{C}}_{i}=\left\{\begin{array}{c} \frac{1}{d-1}\sum_{l=1}^{d-1}{\hat{x}}_{l,d-l}\;\;\;\;\;\;\;\;\;\;\;\;\;\;\;\;\;for\;2\leq d\leq m-1\\ \frac{1}{m}\sum_{l=1}^{m}{\hat{x}}_{l,d-l}\;\;\;\;\;\;\;\;\;\;\;\;\;\;\;\;\;\;\;\;for\;m\leq d\leq L+1\\ \frac{1}{m+L-d+1}\sum_{l=d-L}^{m}{\hat{x}}_{l,d-l}\;\;\;\;\;for\;L+2\leq d\leq m+L \end{array}\right.$$

where ${\hat{x}}_{i,j}$ is the *i*-th row and *j*-th column element of the submatrix $X_{i}$.

**Figure 3:**
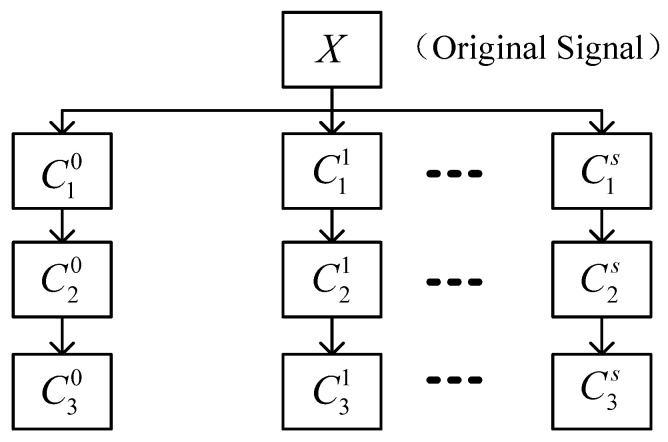
Schematic diagram of ISVDP.

In order to further suppress the model-mixing effect and improve the feature-extraction ability of bearing faults, this paper selects sub-signals of the same frequency by measuring the similarity between sub-signals. The similarity measure function based on the Pearson correlation coefficient is

(10) $$r_{yz}=\frac{\sum_{n=1}^{N}\left(y\left(n\right)-\bar{y}\right)\left(z\left(n\right)-\bar{z}\right)}{\sqrt{\sum_{n=1}^{N}{\left(y\left(n\right)-\bar{y}\right)}^{2}{\left(z\left(n\right)-\bar{z}\right)}^{2}}}$$

where $\bar{y}$ and $\bar{z}$ are the mean values of y(n) and z(n), respectively. According to Equation (10), the related reconstructed sub-signal component is ${\hat{C}}_{i},i=1,\cdots ,m$ the related reconstructed sub-signal component is $C_{i},i=1,\cdots ,s$, and s,s≤m is the number of sub-signals. The sub-signal $C_{i}$ selected by the Pearson correlation coefficient mainly contains one frequency. Therefore, when the decomposition level is J≥2, each sub-signal $C_{i}$ retains only the first sub-signal after decomposition. The above process is repeated for each sub-signal $C_{i}$ according to the decomposition layer *J*. The decomposition structure of ISVDP is shown in Figure 3.

### 3.3. Parameter Selection of ISVDP

The proposed ISVDP includes two preset parameters, namely the number of rows of the Hankel matrix and the number of decomposition layers. If the number of rows of the Hankel matrix is greater than or equal to the number of resonance frequency bands of bearing signals for the same number of decomposition layers, more rows leads to better ability to extract the resonance frequency bands of bearing signals, but the aggregation becomes worse. When the number of rows of the matrix is the same, more decomposition layers leads to a narrower frequency band of the bearing signal, but there is a risk of losing effective features. Therefore, according to reference [27] and practical experience, the range of Hankel matrix rows is from 4 to 20, and the number of decomposition layers is from 3 to 10.

### 3.4. Application of ISVDP in Fault Diagnosis

**Figure 4:**
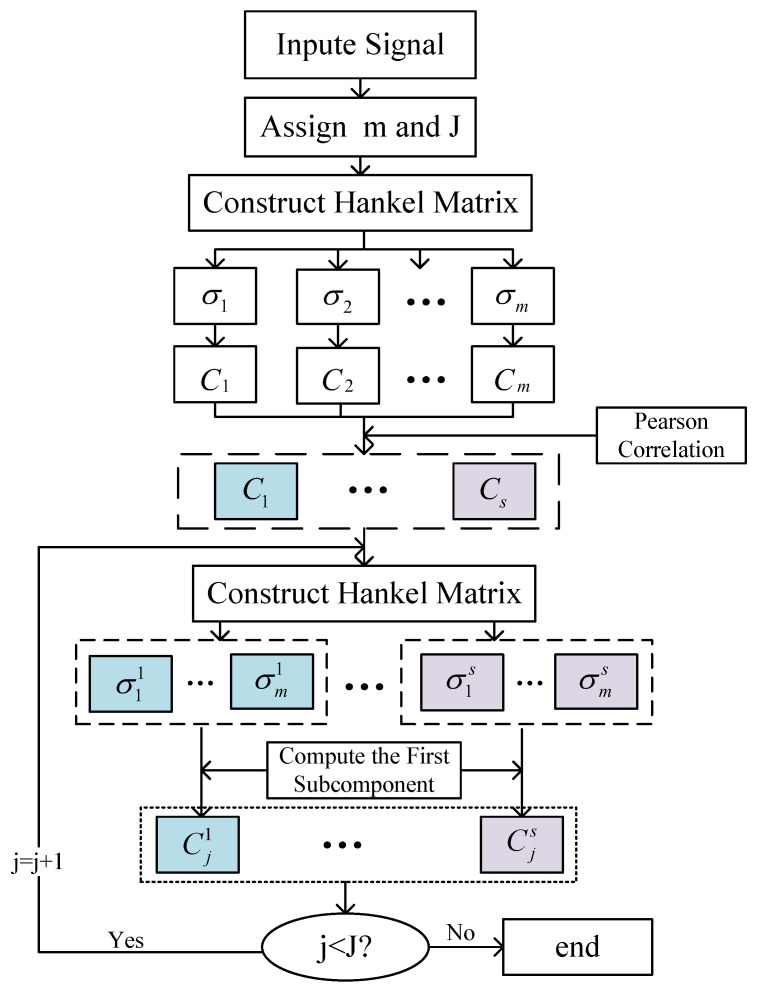
Flowchart of ISVDP on bearing fault diagnosis.

ISVDP decomposes the bearing fault signal containing multiple resonance bands into multiple sub-signals and performs envelope analysis on each sub-signal to realize the fault diagnosis of the bearing by combining the envelope diagram of the sub-signal. The process flow diagram of ISVDP is shown in Figure 4.

## 4. Simulation Analysis

### 4.1. Simulation Parameters

In order to verify the effectiveness of ISVDP in bearing diagnosis, we implemented the bearing fault signal model of Reference [27]. The simulation model of a bearing fault signal is

(11) $$x\left(t\right)=x_{1}\left(t\right)+x_{2}\left(t\right)+x_{3}\left(t\right)+x_{4}\left(t\right)+\xi \left(t\right)$$

where the component signals $x_{j}\left(t\right),j=1,2,3$ are the bearing outer ring fault signal, the connecting shaft signal connected to the bearing, and the external impact signal. The component signals $x_{j}\left(t\right),j=1,2,3$ are

(12) $$x_{j}\left(t\right)=\sum_{n=0}^{M_{j}-1}B_{ij}e^{-\beta_{j}(t-\sum_{i=0}^{n}\Delta T_{ij})}\cos[2\pi f_{R,j}\left(t-\sum_{i=0}^{n}\Delta T_{ij})\right]u(t-\sum_{i=0}^{n}\Delta T_{ij})$$

where $B_{ij}$ is the amplitude of the *i*-th fault shock of the signal $x_{j}\left(t\right)$, $\beta_{j}$ is the structural attenuation coefficient, $f_{R,j}$ is the resonance frequency of the signal $x_{j}\left(t\right)$, $M_{j}$ is the number of shocks in the signal $x_{j}\left(t\right)$, u(t) is the unit step function, $\Delta T_{ij}$ is the interval between the i−1-th and *i*-th shocks of the signal $x_{j}\left(t\right)$—where the impact interval $\Delta T_{ij}$ of the first component signal $x_{1}\left(t\right)$ and the second component signal $x_{2}\left(t\right)$ obeys Gaussian distribution, that is $\left\{\Delta T_{ij}\right\}∼N\left(T_{p,j},\sigma_{\Delta ,j}^{2}\right)$—$T_{p,j}=f_{j}^{-1}$ is the mean, $f_{j}$ is the feature frequency of bearing fault, $\sigma_{\Delta ,j}$ is the standard deviation of time interval fluctuation, and $\sigma_{\Delta ,j}/T_{p,j}\;<2\%$. The impact interval $\Delta T_{ij}$ of the third component signal $x_{3}\left(t\right)$ is $\Delta T_{13}=0.2$ s, $\Delta T_{23}=0.8$ s. The other simulation parameters are shown in Table 1. The harmonic interference signal $x_{4}\left(t\right)$ is

(13) $$x_{4}\left(t\right)=0.2\sin\left(4\pi f_{r}t\right)+0.1\sin\left(6\pi f_{r}t\right)+0.1\sin\left(8\pi f_{r}t\right)$$

where $f_{r}=10.29$ Hz is the harmonic interference frequency; ξ(t) is white Gaussian noise with a standard deviation of 0.22. The sampling frequency is 10 KHz.

FESVDP is a fast decomposition algorithm of ESVDP, so this paper uses SVDP, FESVDP, Empirical Mode Decomposition (EMD), Variational Mode Decomposition (VMD) and ISVDP to process the simulation signal x(t). To verify the effectiveness of the method in this paper, the number of decomposition layers and the number of Hankel matrix rows of FESVDP in [27] are used. There are 3 decomposition layers and 16 Hankel matrix rows. The parameters of EMD and VMD are the default values of the functions in MATLAB.

### 4.2. Results Analysis

**Figure 5:**
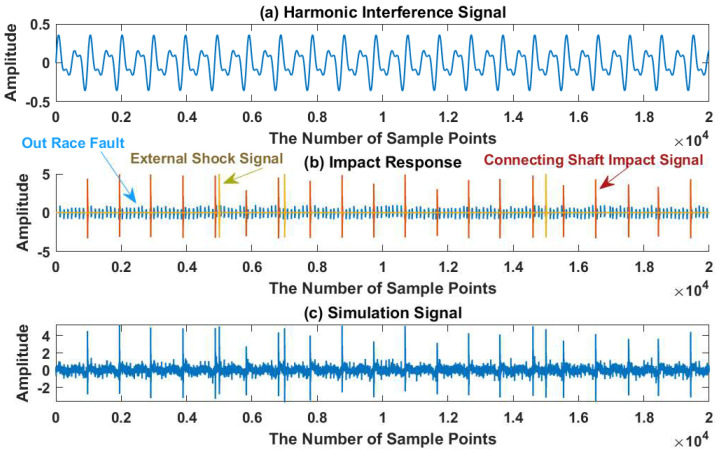
The simulated signal: (**a**) harmonic interference; (**b**) impulse responses excited by defects in the bearing; (**c**) the synthesis signal.

**Figure 6:**
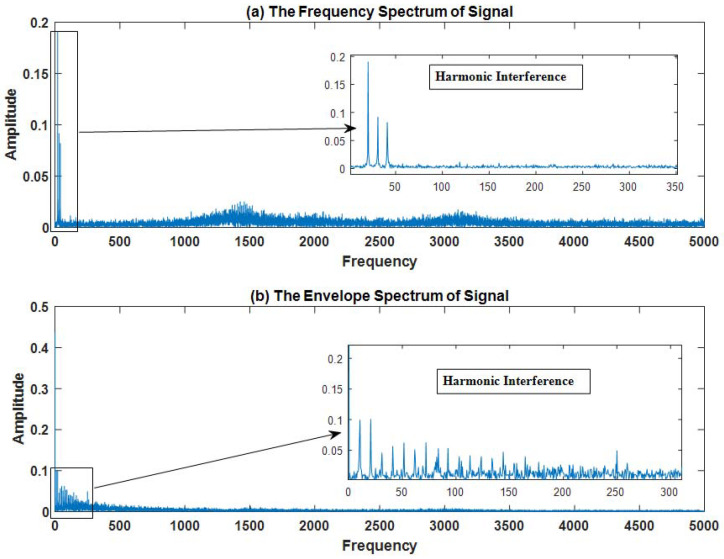
The simulated signal: (**a**) frequency spectrum; (**b**) envelope spectrum.

The waveform diagram of signal x(t) is shown in Figure 5, and the spectrum diagram and envelope diagram of signal x(t) are shown in Figure 6. In the Fourier spectrum shown in Figure 6a, the resonance band centered at $f_{R,j},j=1,2,3$ is not obvious. In the envelope spectrum shown in Figure 6b, the feature frequency $f_{1}=83.33$ Hz of the bearing fault is covered by other peaks.

**Figure 7:**
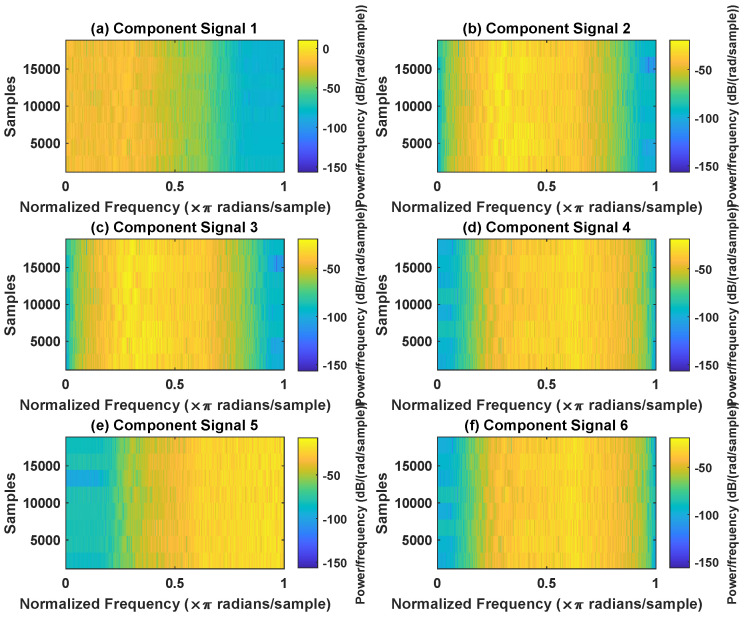
Decomposition results of SVDP with m=16 and J=3: (**a**–**f**) time–frequency diagram of 1st to 6th subcomponents, respectively.

**Figure 8:**
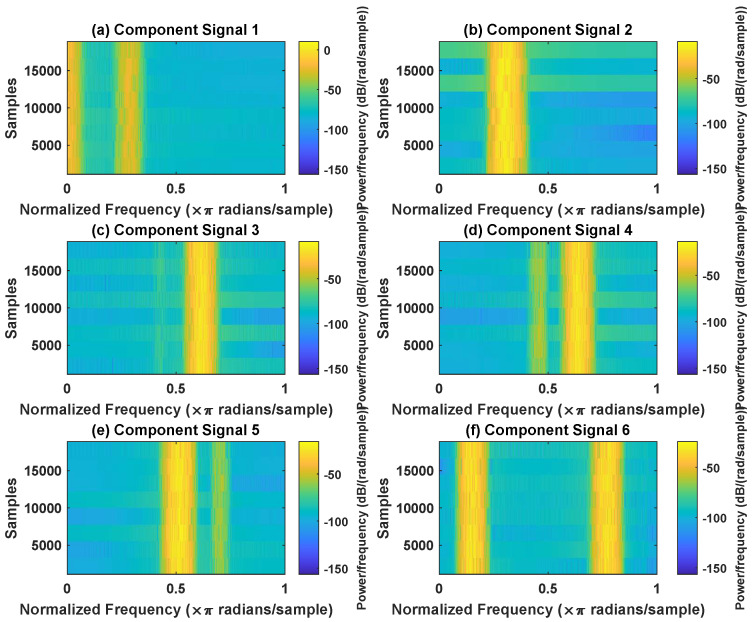
Decomposition results of FESVDP with m=16 and J=3: (**a**–**f**) time–frequency diagram of 1st to 6th subcomponents, respectively.

**Figure 9:**
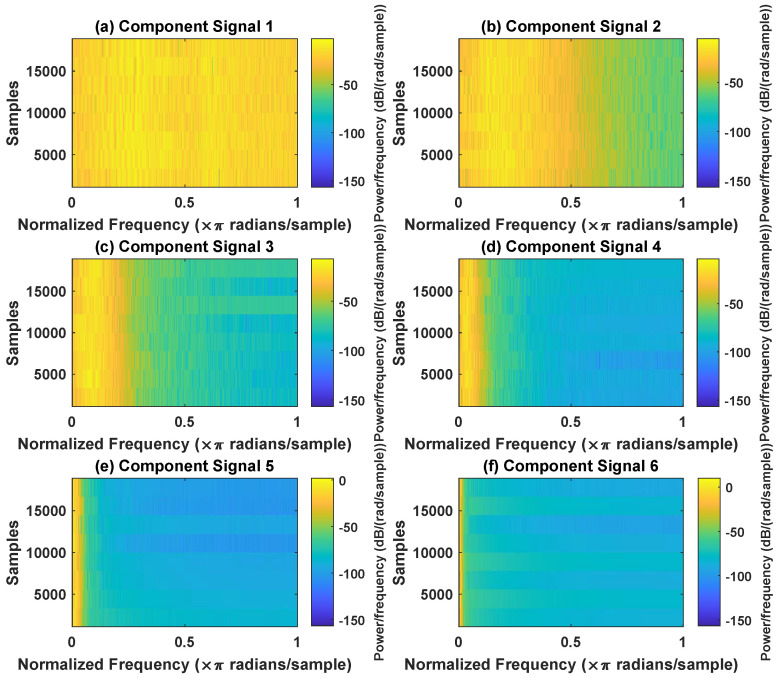
Decomposition results of EMD with m=16: (**a**–**f**) time–frequency diagram of 1st to 6th subcomponents, respectively.

**Figure 10:**
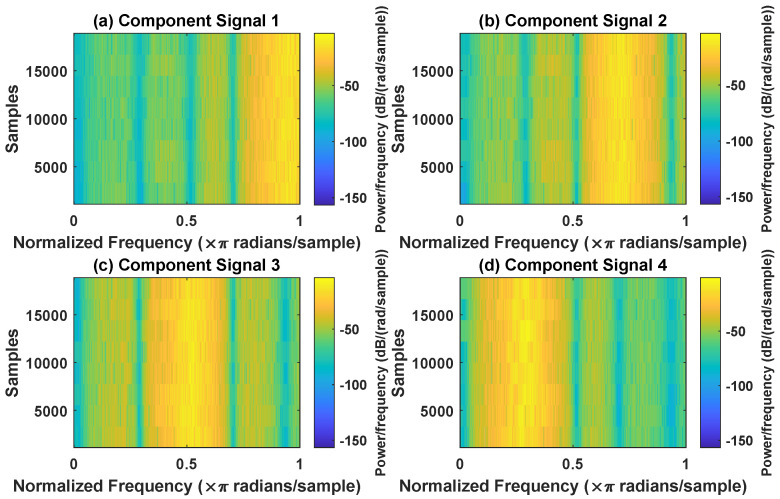
Decomposition results of VMD with m=16: (**a**–**d**) time–frequency diagram of 1st to 4th subcomponents, respectively.

**Figure 11:**
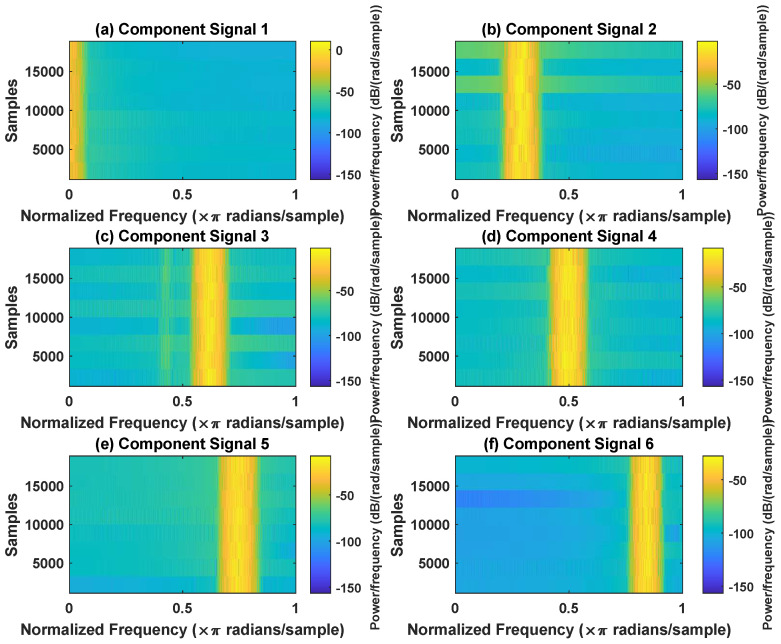
Decomposition results of ISVDP with m=16 and J=3: (**a**–**f**) time–frequency diagram of 1st to 6th subcomponents, respectively.

In order to effectively express the decomposition effect of each algorithm and save the layout of the article, the first six component signals after the SVDP, FESVDP, EMD and ISVDP decomposition simulation signals and the first four component signals after VMD decomposition simulation signals are selected to make the time–frequency domain diagram and envelope diagram, respectively, of the component signal. Figure 7, Figure 8, Figure 9, Figure 10 and Figure 11 are the time–frequency diagrams of the component signals after the simulation signal is decomposed by SVDP, FESVDP, EMD, VMD and ISVDP, respectively. It can be seen that compared with Figure 8 and Figure 11, the frequency band in each component signal in Figure 7, 9 and Figure 10 are wider, and multiple component signals contain the same frequency components, which indicates that SVDP cannot effectively extract signal features. From Figure 8a,f, it can be clearly seen that although the frequency bands in each component signal after FESVDP decomposition are narrow, there are two frequency bands in the same component signal, so there is still mode mixing after FESVDP decomposition of the bearing signal. From Figure 11, it can be seen that the frequency band of each component signal after ISVDP decomposition is narrow and one component signal contains only one frequency band. Therefore, ISVDP can effectively extract signal features and effectively suppress modal mixing.

**Figure 12:**
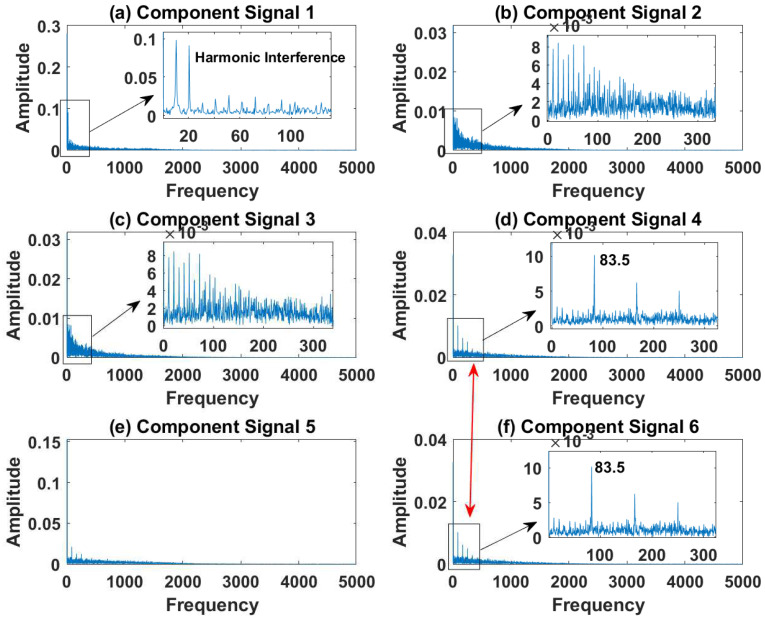
Decomposition results of SVDP with m=16 and J=3: (**a**–**f**) envelop of 1st to 6th subcomponents, respectively.

**Figure 13:**
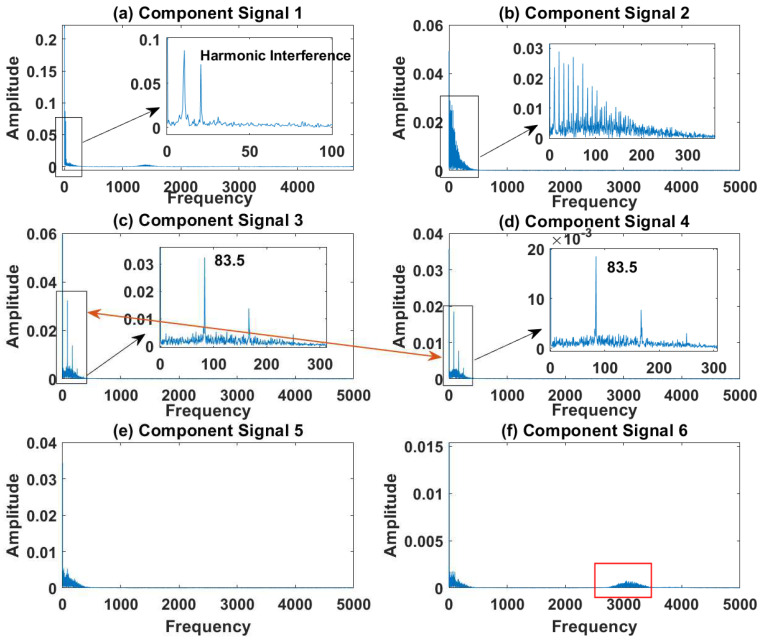
Decomposition results of FESVDP with m=16 and J=3: (**a**–**f**) envelop of 1st to 6th subcomponents, respectively.

**Figure 14:**
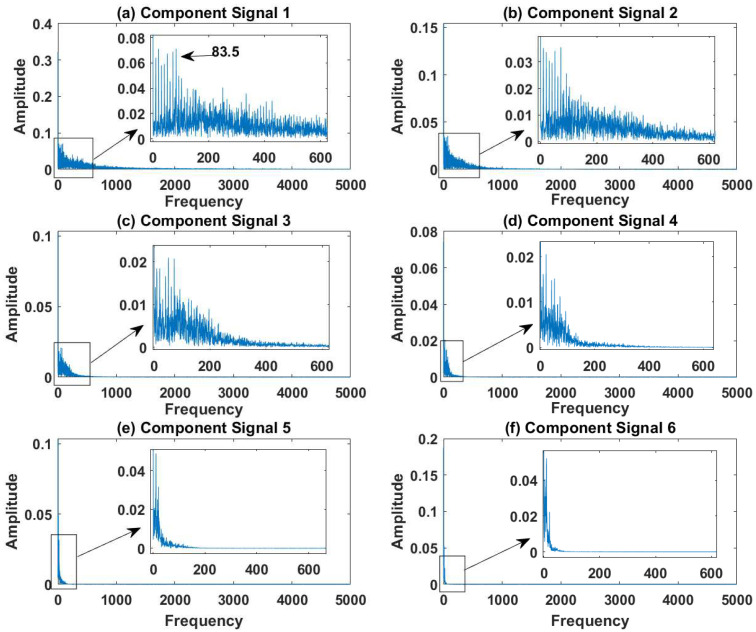
Decomposition results of EMD with m=16: (**a**–**f**) envelop of 1st to 6th subcomponents, respectively.

**Figure 15:**
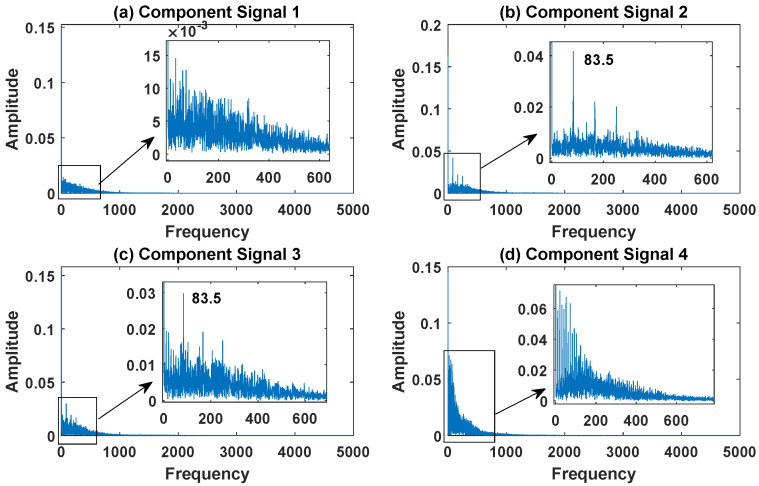
Decomposition results of VMD with m=16: (**a**–**d**) envelop of 1st to 4th subcomponents, respectively.

**Figure 16:**
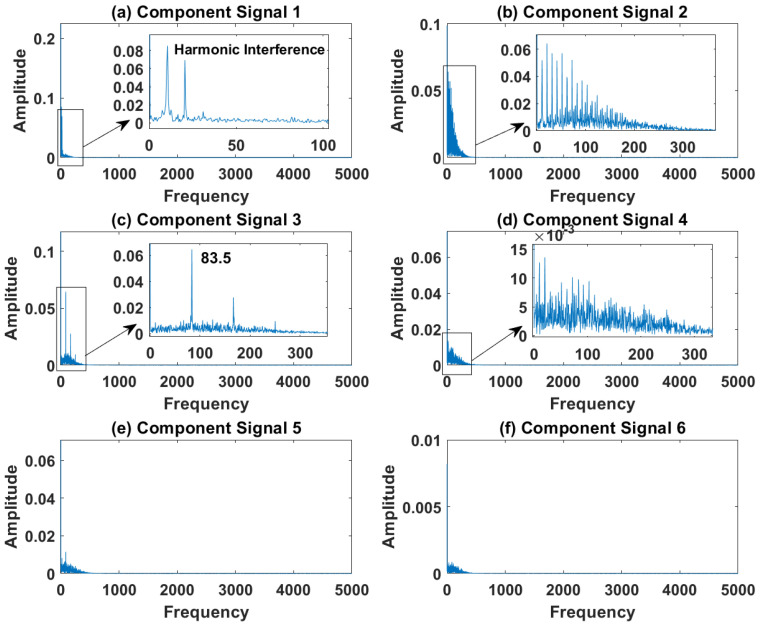
Decomposition results of ISVDP with m=16 and J=3: (**a**–**f**) envelop of 1st to 6th subcomponents, respectively.

Figure 12, Figure 13, Figure 14, Figure 15 and Figure 16 are the envelopes of the sub-signals after SVDP, FESVDP, EMD, VMD and ISVDP decomposition, respectively. It can be seen that the frequency bands in the envelope spectrum of FESVDP and ISVDP are narrower compared to SVDP, EMD and VMD, which corresponds to the time–frequency spectrum. Figure 12a, Figure 13a and Figure 16a are the envelope diagrams of harmonic interference signals. It can be seen that SVDP, FESVDP and ISVDP can effectively separate harmonic signals. However, it is difficult to distinguish the characteristic frequency of harmonic interference signals in Figure 14 and Figure 15. Therefore, EMD and VMD are difficult to effectively separate harmonic signals. In addition, it can be seen from Figure 12d,f, Figure 13c,d, Figure 15b,c and Figure 16c are envelope diagrams of bearing faults. It can be seen that the Four methods can effectively decompose the characteristic frequency $f_{1}=83.33$ Hz of bearing faults, but EMD is difficult to effectively extract the characteristic frequency of bearing fault. However, the envelope spectra of Figure 12d,f contain fault characteristic frequencies, the envelope spectra of Figure 13c,d contain fault characteristic frequencies, the envelope spectra of Figure 15b,c contain fault characteristic frequencies and the envelopes of Figure 13a,f are mixed with other frequency information, which indicates that both SVDP, FESVDP and VMD have mode mixing. This is because when SVDP iteratively decomposes, each signal can only get two sub-signals, and the signal information is mainly concentrated in the approximate signal, resulting in sub-signals with the same frequency appearing in different sub-signal components. The FESVDP is due to the combination of two adjacent sub-signal components with different frequencies, which artificially causes mode mixing. VMD is due to the small number of decomposed modes, which causes the VMD to decompose the signal insufficiently, resulting in mode aliasing.

## 5. Experimental Verification

### 5.1. Data Description

The experimental drive end bearing data of Case Western Reserve University in the United States was used [30], and the test bearing was SKF6205-2RS deep groove ball bearing. The mixed signal is composed of two inner race fault data. The sampling frequency is 12 KHz. To further weaken the fault feature, 13 dB noise is superimposed on the mixed signal. The parameters of the inner race fault are shown in Table 2. The number of decomposition layers and the number of rows of the Hankel matrix of SVDP, FESVDP and ISVDP are 3 and 16, respectively. The parameters of EMD and VMD are the default values of the functions in MATLAB.

### 5.2. Test Results and Analysis

**Figure 17:**
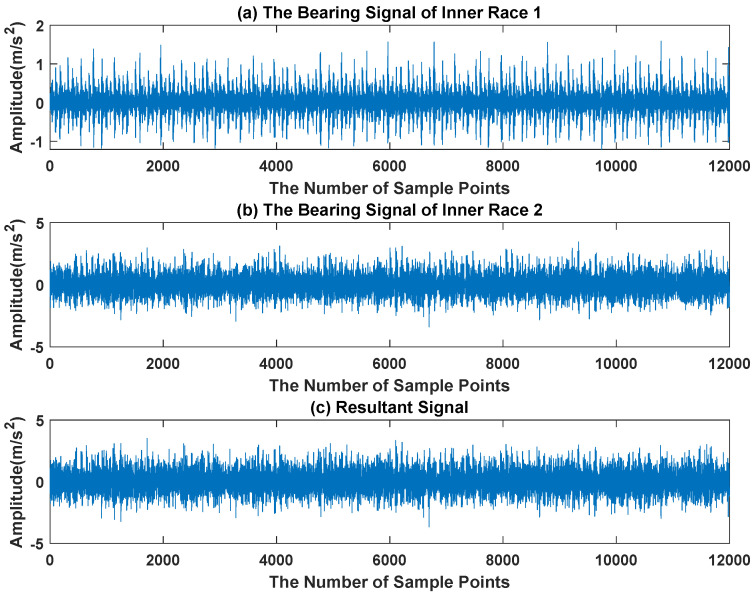
The Waveform of Composite Signal.

**Figure 18:**
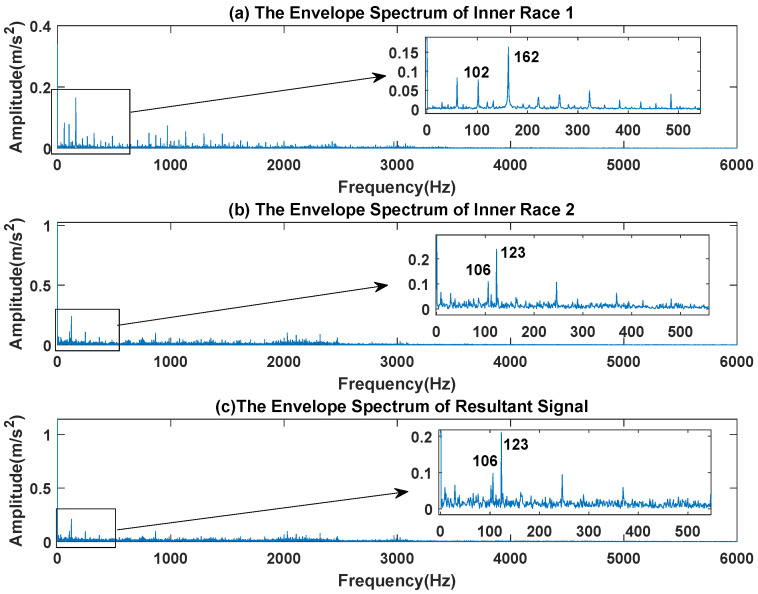
The Envelope Diagram of Composite Signal.

The waveform diagram and envelope diagram of the mixed signal are shown in Figure 17 and Figure 18. The main frequencies of inner race fault 1 and inner race fault 2 are 102 Hz, 162 Hz, 106 Hz and 123 Hz respectively. It can be seen from Figure 18c that the envelope peak of inner race fault 1 is almost covered by inner race fault 2.

**Figure 19:**
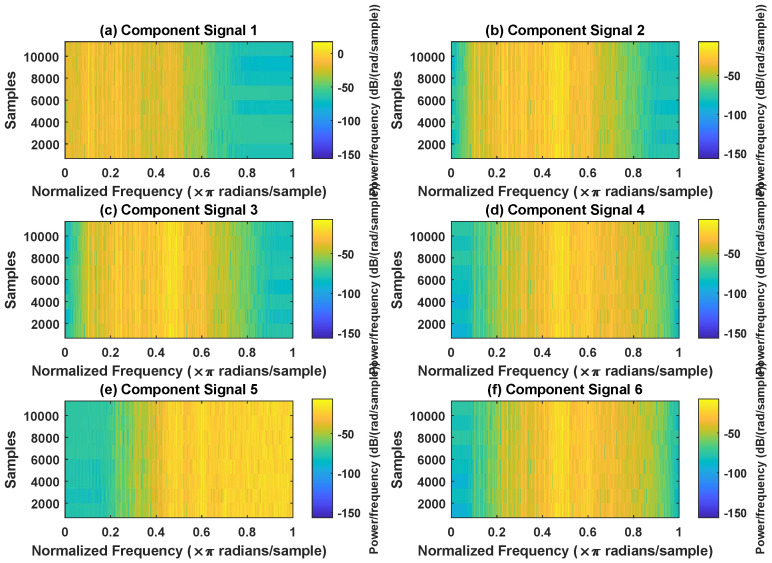
Decomposition results of SVDP with m=16 and J=3: (**a**–**f**) time–frequency diagram of 1st to 6th subcomponents, respectively.

**Figure 20:**
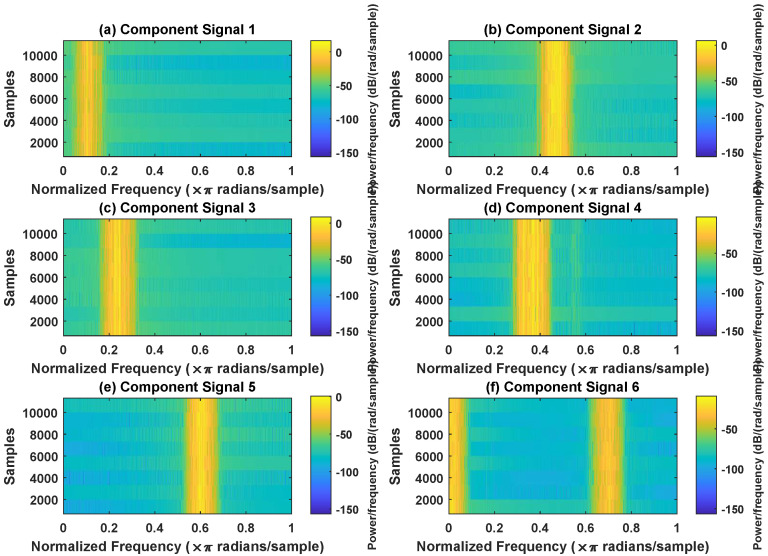
Decomposition results of FESVDP with m=16 and J=3: (**a**–**f**) time–frequency diagram of 1st to 6th subcomponents, respectively.

**Figure 21:**
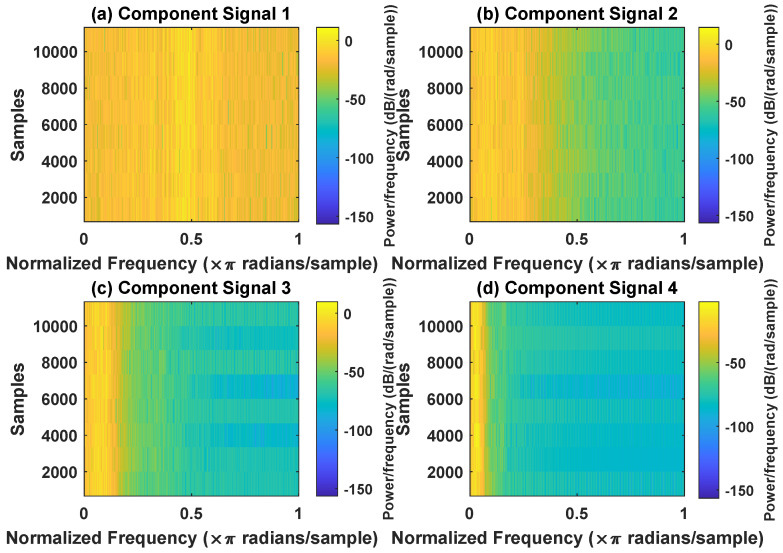
Decomposition results of EMD with m=16: (**a**–**d**) time–frequency diagram of 1st to 4th subcomponents, respectively.

**Figure 22:**
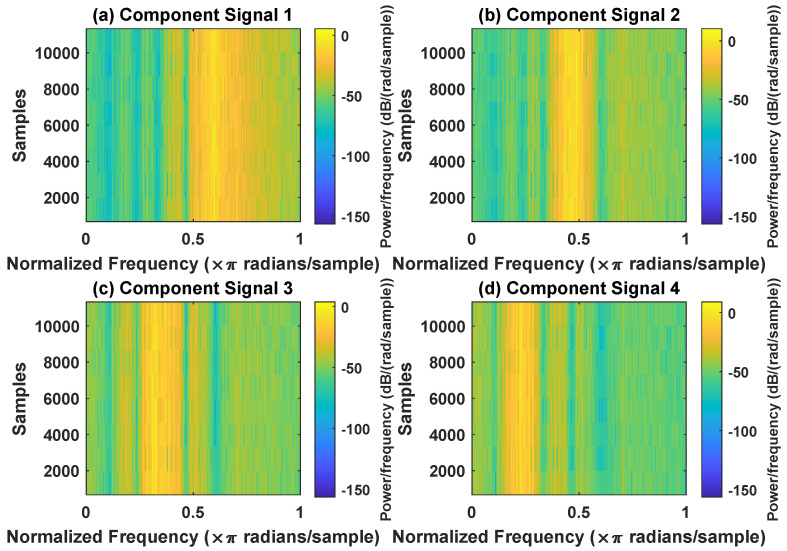
Decomposition results of VMD with m=16: (**a**–**d**) time–frequency diagram of 1st to 4th subcomponents, respectively.

**Figure 23:**
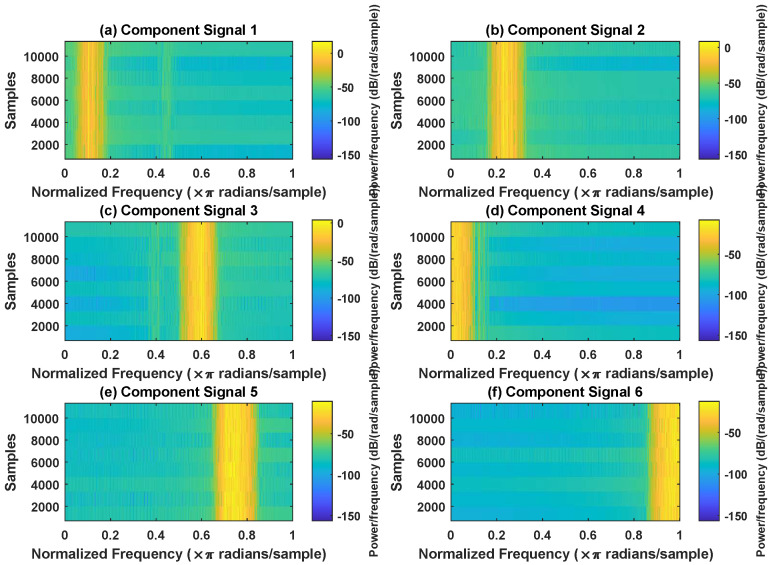
Decomposition results of ISVDP with m=16 and J=3: (**a**–**f**) time–frequency diagram of 1st to 6th subcomponents, respectively.

In order to effectively express the decomposition effect of each algorithm and save the layout of the article, the first six component signals after the SVDP, FESVDP, and ISVDP decomposition simulation signals and the first four component signals after EMD and VMD decomposition simulation signals are selected to make the time–frequency domain diagram and envelope diagram of the component signal respectively. Figure 19, Figure 20, Figure 21, Figure 22 and Figure 23 are the time–frequency diagrams of the sub-components after SVDP, FESVDP, EMD, VMD and ISVDP decomposition, respectively. From Figure 19, Figure 21 and Figure 22, it can be seen that the frequency band of each component signal after SVDP, EMD and VMD decomposition is wider, and multiple frequency bands are distributed in different component signals. This shows that SVDP, EMD and VMD algorithms are difficult to effectively extract bearing fault features, and there is modal mixing. However, in Figure 20 and Figure 23, the frequency band of each component signal decomposed by FESVDP and ISVDP is narrow, therefore, both FESVDP and ISVDP can extract bearing fault features better. Besides, it can be clearly seen from Figure 20f that the component signal after the FESVDP bearing signal contains two frequency bands. Therefore, there is still modal mixing after the FESVDP decomposes the bearing signal. However, the above phenomenon does not appear in Figure 23, indicating that ISVDP can effectively extract signal features, and can effectively suppress the modal mixing phenomenon.

**Figure 24:**
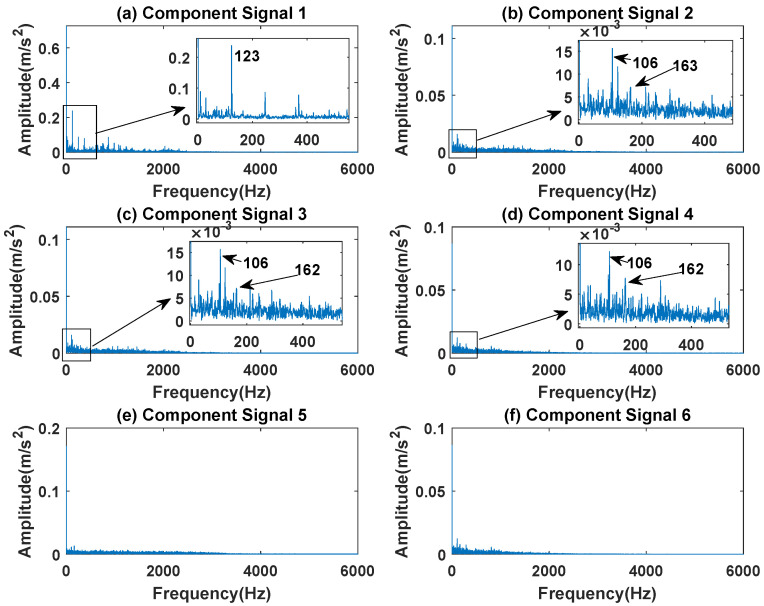
Decomposition results of SVDP with m=16 and J=3: (**a**–**f**) envelop of 1st to 6th subcomponents, respectively.

**Figure 25:**
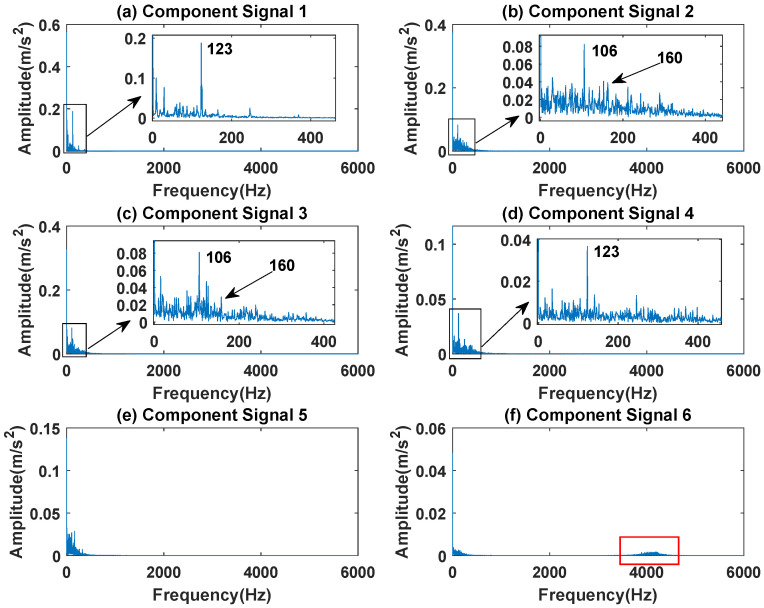
Decomposition results of FESVDP with m=16 and J=3: (**a**–**f**) envelop of 1st to 6th subcomponents, respectively.

**Figure 26:**
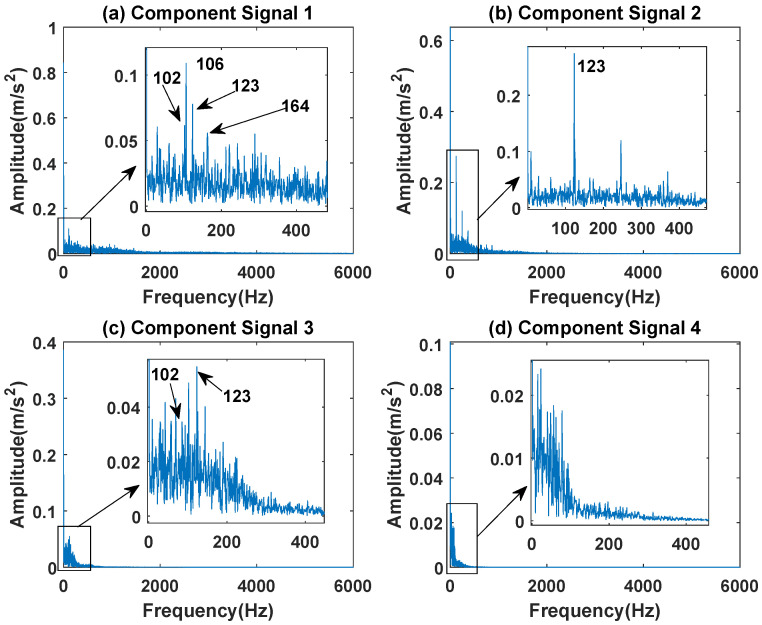
Decomposition results of EMD with m=16: (**a**–**d**) envelop of 1st to 4th subcomponents, respectively.

**Figure 27:**
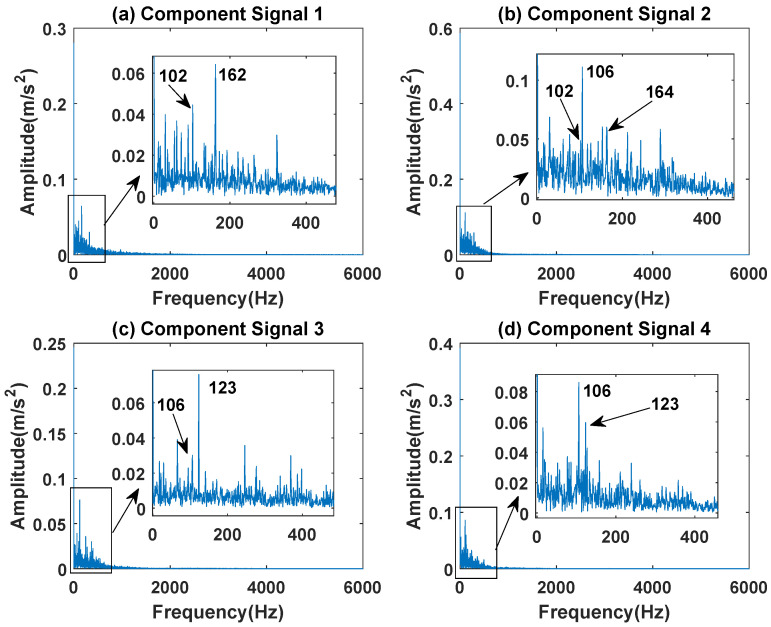
Decomposition results of VMD with m=16: (**a**–**d**) envelop of 1st to 4th subcomponents, respectively.

**Figure 28:**
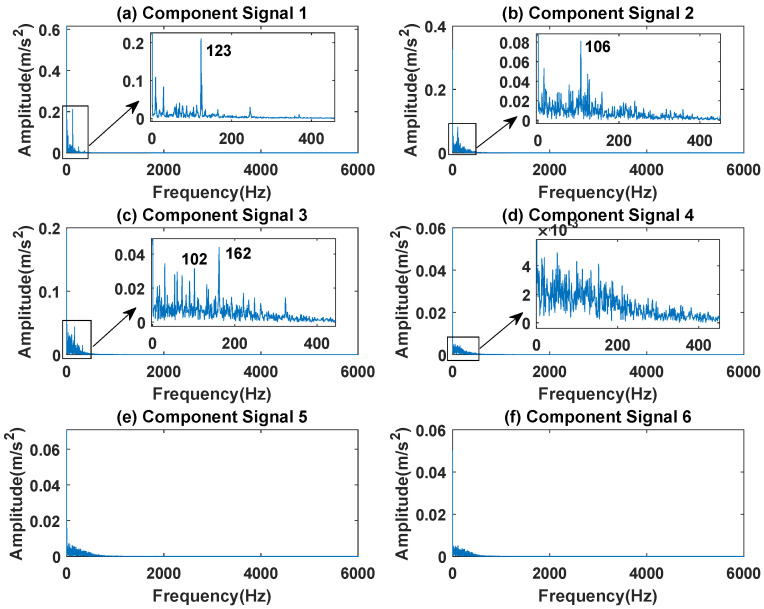
Decomposition results of ISVDP with m=16 and J=3: (**a**–**f**) envelop of 1st to 6th subcomponents, respectively.

Figure 24, Figure 25, Figure 26, Figure 27 and Figure 28 are the envelope diagrams of the sub-components after SVDP, FESVDP, EMD, VMD and ISVDP decomposition, respectively. It can be seen that the spectral peaks in the envelope spectra of FESVDP and ISVDP are narrower compared to SVDP, EMD and VMD, which corresponds to the time–frequency diagram. In Figure 24b–d, Figure 25b,c, Figure 26a,c and Figure 27b the characteristic frequency of inner race fault 1 and the characteristic frequency of inner race fault 2 are in the same envelope diagram. This shows that SVDP, FESVDP, EMD and VMD cannot effectively separate inner race fault 1 and inner race fault 2. In addition, it can be seen from Figure 27 that although compared with SVDP, FESVDP and EMD, VMD can relatively effectively separate inner race fault 1 and inner race fault 2, and has a relatively good ability to extract bearing fault features, but the characteristic frequency of inner race fault 2 appears in the two separated signals after VMD decomposition. Therefore, SVDP, FESVDP, EMD and VMD can not effectively separate inner race fault 1 and inner race fault 2 and there is modal mixing in the decomposed component signals. However, from Figure 28, it can be seen that ISVDP can effectively separate the inner race fault 1 and the inner race fault 2, and the separated features do not appear in the two separate signals after ISVDP decomposition. Therefore, ISVDP has better bearing fault feature extraction ability and can effectively suppress modal nixcing.

In summary, the results of the five algorithms in simulation and experiment are shown in Table 3. It can be seen that the ISVDP proposed in this paper can extract bearing fault features more effectively and suppresses modal aliasing, while SVDP and EMD are the worst in extracting bearing fault features and suppressing modal aliasing.

## 6. Conclusions

In this paper, in order to suppress the modal-aliasing phenomenon existing in the SVDP extraction of bearing signal fault features and to improve the feature extraction ability of SVDP, an improved singular value decomposition package algorithm is proposed. The feature extraction ability of SVDP is improved by changing the structure of the Hankel matrix in SVDP and using similarity to select signal sub-components, and the modal-aliasing phenomenon is effectively suppressed. The effectiveness of the ISVDP method is verified by simulations and experiments. The results show that ISVDP can effectively extract fault features. Compared with SVDP and FESVDP, ISVDP has the following advantages: (1) ISVDP can effectively suppress the model-mixing phenomenon; (2) ISVDP can extract fault features more effectively and improve the accuracy of fault diagnosis.

ISVDP can extract the fault features of bearing signals without relying on prior knowledge and can effectively suppress modal aliasing. However, ISVDP needs to determine the number of rows of the Hankel matrix and the number of decomposition layers, especially the number of rows of the Hankel matrix. Since the process of signal conversion into the Hankel matrix in ISVDP is the same as that of signal conversion into a trajectory matrix in singular spectrum analysis, it is one of the future research methods of ISVDP to determine the number of rows of the Hankel matrix by referring to the method of determining the dimensions of the trajectory matrix in singular spectrum analysis.

## Figures and Tables

**Table 1:** Parameters of the simulation signal.

*j*	$B_{\mathrm{ij}}$	$\beta_{j}$	$f_{R,j}$ (Hz)	$T_{p,j}$ (s)	$M_{j}$	$\sigma_{\Delta ,j}/T_{p,j}\;$
1	1	1000	3100	0.012	167	1%
2	5	1200	1400	0.097	21	0.5%
3	5	1200	2000	-	3	-

**Table 2:** Structural parameters of rolling bearing.

Status	Fault Size of Samples	Motor Load	Approx. Motor Speed
Inner Race 1	0.1778 (mm)	0 (HP)	1797 (rpm)
Inner Race 2	0.7112 (mm)	3 (HP)	1730 (rpm)

**Table 3:** Comparison of the results of five algorithms in simulations and experiments.

	Simulation	Experiment
SVDP	1. Reduce harmonic interference; 2. Extract fault features; 3. Mode mixing	1. Inner race faults 1 and 2 cannot be separated; 2. Mode mixing
FESVDP	1. Reduce harmonic interference; 2. Extract fault features; 3. Mode mixing	1. Inner race faults 1 and 2 cannot be separated; 2. Mode mixing
EMD	1. Harmonic interference exists; 2. Fault features cannot be extracted; 3. Mode mixing	1. Inner race faults 1 and 2 cannot be separated; 2. Mode mixing
VMD	1. Harmonic interference exists; 2. Extract fault features; 3. Mode mixing	1. Partial separation of inner race faults 1 and 2; 2. Mode mixing
ISVDP	1. Reduce harmonic interference; 2. Extract fault features; 3. Restrain modal mixing	1. Effective separation of inner race faults 1 and 2; 2. Restrain modal mixing

## Data Availability

Not applicable.

## Abbreviations

The following abbreviations are used in this manuscript:

SVDP	singular value decomposition package
ISVDP	improved singular value decomposition package
SVD	singular value decomposition
MSVD	multi-resolution singular value decomposition
ESVDP	extended singular value decomposition package
FESVDP	fast extended singular value decomposition package
